# Characterisation of *Clostridium difficile* Biofilm Formation, a Role for Spo0A

**DOI:** 10.1371/journal.pone.0050527

**Published:** 2012-12-07

**Authors:** Lisa F. Dawson, Esmeralda Valiente, Alexandra Faulds-Pain, Elizabeth H. Donahue, Brendan W. Wren

**Affiliations:** Department of Pathogen Molecular Biology, London School of Hygiene and Tropical Medicine, London, United Kingdom; Institute Pasteur, France

## Abstract

*Clostridium difficile* is a Gram-positive anaerobic, spore-forming bacillus that is the leading cause of nosocomial diarrhoea worldwide. We demonstrate that *C. difficile* aggregates and forms biofilms *in vitro* on abiotic surfaces. These polymicrobial aggregates are attached to each other and to an abiotic surface by an extracellular polymeric substance (EPS). The EPS matrix provides the scaffold bonding together vegetative cells and spores, as well as forming a protective barrier for vegetative cells against oxygen stress. The master regulator of sporulation, Spo0A, may play a key role in biofilm formation, as genetic inactivation of *spo0A* in strain R20291 exhibits decreased biofilm formation. Our findings highlight an important attribute of *C. difficile* pathogenesis, which may have significant implications for infection, treatment and relapse.

## Introduction

*Clostridium difficile* is an important nosocomial infection, responsible for outbreaks of potentially fatal diarrhoea worldwide. The disease spectrum of *C. difficile* infection (CDI) ranges from mild self-limited illness to severe life-threatening pseudomembranous colitis [1]. Since 2000, there has been a dramatic increase in rates and severity of CDI particularly in North America and Europe [2]–[4]. The population at risk for CDI includes not only patients on antimicrobial and other therapies that can alter the balance of the gut microbiota [5], but also the immune-compromised and elderly. Relapse or re-infection of *C. difficile* in susceptible individuals is also prevalent [6], 15–35% of patients relapse within the first 2 months post-treatment [7]. The reason for persistence of *C. difficile* within the host and the cause of relapse is poorly understood.

For many pathogenic bacteria, the formation of biofilms is linked to survival, persistence, resistance to antimicrobials, colonization and disease [8]. A biofilm is defined as polymicrobial aggregates attached to each other and/or to surfaces [9]. They are found in a wide variety of settings, such as on medical devices (catheters) [10], [11], in water and sewage pipes [12], food processing [13] and treatment of wastewater in oil refineries [14]. Biofilms are also an important factor in human disease [9], with up to 80% of bacterial infections linked to biofilms [11]. Consequently biofilms can have a detrimental impact on both health and economics. Many pathogenic bacteria form biofilms to provide a physical barrier to external stresses, such as desiccation, antimicrobials and biocides, as well as to evade the host immune response, preserve nutrients and retain mechanical integrity [8], [15] [16]. In the spore forming aerobe *Bacillus subtilis* it has been shown that biofilm formation is an important lifestyle choice for survival. There are three pathways differentially regulated by the sporulation master regulator Spo0A, which determine cell fate; sporulation, matrix formation or cannibalism [17]–[20]. The formation and maintenance of biofilms in *B. subtilis* is regulated by matrix producing cells, combined with the sacrifice of a subpopulation of bacteria by cannibalism toxins to provide nutrients for the biofilm community and form part of the biofilm structure [21], [22]. The matrix production and cannibalism phenotypes delay the commitment to enter the sporulation pathway [23]. *C. difficile* possesses a Spo0A orthologue with 56% amino acid identity to that of *B. subtilis*, this orthologue has been shown to be essential to the initiation of sporulation in *C. difficile* [24].

The advantage of a biofilm lifestyle is; self-preservation, enabling the bacteria to survive and colonize a particular niche [8] or hostile environment. One key component of the biofilm is the extracellular polymeric substance (EPS), which forms the matrix, in which the bacteria are encased, and provides the scaffold by which bacteria adhere to each other and to surfaces. The composition of the EPS is strain dependent, but the majority are comprised of polysaccharides, nucleic acids, lipids and proteins, which are secreted by biofilm-producing bacteria [25]. As well as providing a protective environment from external influences, the EPS matrix can also provide a nutrient source, from the recycling of lysed cells [22], and can immobilise cells in close proximity to facilitate interactions and cell-cell communication [8].

In this study we establish that *C. difficile* aggregates to form biofilms *in vitro* on abiotic surfaces. The EPS matrix provides the scaffold for both viable vegetative cells and spores within the biofilm, which provides protection from oxygen stress. Gene inactivation of *spo0A* significantly reduced both biofilm formation and protection against oxygen stress, suggesting a role for Spo0A in the induction of biofilm formation in *C. difficile*.

## Materials and Methods

### Growth of bacterial strains

*C. difficile* strains used in this study are summarised in table 1 [24], [26]–[28]. Strains were stored at −80°C and were cultured on Brazier's agar (Bioconnections, UK) plus 4% egg-yolk, 250 µg/ml Cycloserine, 8 µg/ml Cefoxitin (Bioconnections) and 1% defibrinated horse blood (TCS biosciences), or on BHIS agar comprised of brain heart infusion media (Oxoid) supplemented with 0.5% w/v yeast (Sigma) and 0.1% L-Cysteine (Sigma). *Clostridium perfringens* NCTC 8237 strain, the positive control for the crystal violet biofilm formation assay, was cultured on blood agar (Oxoid). Liquid cultures were grown in BHIS broth. All cultures were grown in an anaerobic atmosphere (10% CO_2_, 10% H_2_, 80% N_2_) at 37°C. *Escherichia coli* strains Top10 (Invitrogen) and CA434 [29], the conjugation donor, were grown in Luria-Bertani (LB) broth or agar (Oxiod) supplemented with 12.5 µg/ml Chloramphenicol (Sigma).

**Table 1:** Strains and plasmids used in this study.

Strains	Characteristics	Source
630Δerm	An erythromycin derivative of *C. difficile* 630, PCR ribotype 012	Hussain et al., 2005
R20291	Hypervirulent PCR ribotype 027, isolated from an outbreak in 2004-2004	Stabler et al., 2009
*C. perfringens*	strain NCTC 8237	Peter Donachie
R20291_*spo0A*::CT	R20291 containing *spo0A*::ermB	This study
R20291_*spo0A*::CT::p*spo0A*	R20291_*spo0A*::CT complemented with pRPF101 containing the 825-bp spo0A CDS and promoter	Deakin et al., 2012

Plasmid		
pMTL007cdi_spooA_17	pMTL007 containing spo0A::ermB	Heap et al., 2007
pMTL007CE-2	pMTL007 modified to contain an *fdx* promoter	Heap et al., 2010
pLD_*spo0A*	pMTL007CE-2 containing *spo0A*::ermB	This study
p*spo0A*	pRPF101 containing the 825-bp spo0A CDS and promoter	Provided by Laura Deakin

### Biofilm formation and visualization

*C. difficile* strains were grown on Brazier's agar for 2–3 days. Three single colonies were used to inoculate 10 ml of pre-equilibrated BHIS media in vented tissue culture (TC) flasks (25 cm^3^, Falcon) for primary cultures, which were incubated at 37°C, shaking at 65 rpm for 16 h. These primary cultures were then used to inoculate different vessels (TC flasks, 24 well plates, 24 well plates containing Thermanox coverslips) to assess biofilm formation (all with a 1/10 dilution, starting OD600 0.15+/−0.02). All images were compiled using Adobe Photoshop elements 8.0.

#### a) Attached to abiotic surface – tissue culture flasks

Duplicate 10 ml liquid cultures were set up in BHIS as described in the previous section, using the same primary culture. These were incubated statically for 24 hours, three days or six days, prior to being removed from the anaerobe hood and photographed using a Cannon 600D SLR (mounted on a copy stand with lighting unit (Kaiser RS2) with a 50 mm prime lens). These were performed in triplicate.

#### b) Attached to abiotic surface – Scanning electron microscopy (SEM) and confocal microscopy

- SEM: Duplicate 2 ml liquid cultures (BHIS) were inoculated into pre-equilibrated low evaporation lid 24-well plates (Nunc) containing plastic coated coverslips (Thermanox 13 mm plastic coated coverslips, Fisher), including two media blank controls. The plates were sealed with Nescofilm® and incubated statically at 37°C under anaerobic conditions for three or six days. After which, 1 ml fixative (2.5% Paraformaldehyde/2.5% Glutaraldehyde/0.1M Sodium cacodylate pH 7.4) was added to each well, including the media blank controls. The samples were washed in 0.1 M Na cacodylate, post fixed in 1% osmium tetroxide/0.1 M Na cacodylate, and stored in MilliQ water overnight at 4°C. These were then dehydrated in 30%, 50%, 70%, 80%, 90% and 100% Ethanol and mounted onto an aluminium stub and air-dried prior to sputter coating.
- Confocal microscopy. Strains were grown on Thermanox plastic coated 13 mm coverslips (Fisher) in low evaporation lid 24-well plates as described above, including two media blank controls. After three days and six days of growth the media was removed and the coverslips were washed in the wells with dH_2_O before 200 µl of stain was applied and incubated according to the manufacturers' guidelines. The stains used were FilmTracer™ LIVE/DEAD® biofilm viability Kit (Life Technologies), comprised of Syto9 and Propidium iodide. These coverslips were mounted face up onto glass slides and a coverslip (22 mm×22 mm) was applied over a 30% glycerol mounting media and sealed with clear nail varnish. Slides were visualised under oil immersion (×40 objective) using a laser confocal microscope (Zeiss microscope). Single stains of Syto9 and Propidium iodide were performed to control for any cross bleed between channels, before slides containing combined stains were processed. The excitation/emission was 488 nm/505–550 nm for Syto9 and 543 nm/>650 nm for Propidium iodide, these were collected in tandem for all samples. The assays were performed in quadruplicate. Images were analysed using Zeiss LSM image browser.

#### c) Crystal violet assay; BHIS media was pre-equilibrated for 16 h in low evaporation lid 24-well plates (Nunc International, UK) prior to inoculation, with a 1/10 dilution of the primary cultures (outlined above)

Three media control blanks were included per plate. Bacteria were grown statically in 2 ml of BHIS in 24-well plates (Nunc) for three or six days at 37°C, alongside three unseeded media blank controls. To prevent evaporation, plates were sealed with Nescofilm®. The media was removed by pipetting, after which the wells were washed once with 700 µl 1× PBS prior to incubation for 30 min at room temperature with 700 µl of filter-sterilized 1% (v/v) crystal violet (TCS biosciences). Excess crystal violet was removed from the wells, followed by three washes with 1× PBS. Images were taken of the 24 well plates using a Canon 600D SLR. The crystal violet was extracted by adding 700 µl methanol to each well and incubated for 15 min at room temperature. The OD595 was measured in a Spectrophotometer (Biotek, UK). All assays were performed with a minimum of four biological and three technical replicates. Statistical analysis was performed using Stata/IC 12. A partial F-test was performed to determine if there were strain specific differences in biofilm formation (*p*<0.05 indicates strain specific differences). Regression analyses were performed to determine whether there were any strain specific differences in biofilm formation compared to a reference strain (*p*<0.05) a) strains 630Δ*erm* (reference), R20291 and *C. perfringens* and b) strains R20291 (reference), *spo0A* mutant (R20291_*spo0A*::CT) and complement (R20291_*spo0A*::CT::p*spo0A*). *p*<0.05 indicates a significant link between strain and biofilm formation, and the coefficient (C) determines whether biofilm formation is higher (positive number) or lower (negative number) than the reference (C = 0).

### Production of gene inactivation mutants

A gene inactivation mutant was constructed in the sporulation master regulator Spo0A, using the ClosTron system (table 1) [30], [31] in *C. difficile* strain R20291 [27]. The group II Ll.LtrB intron was retargeted to the gene of interest by SOE PCR as previously described [32], with oligonucleotides (listed in table S1) designed using the Sigma TargeTron website (http://www.sigma-genosys.com/targetron/website). PCR products were cloned into *HindIII* and *BsrGI* sites of pMTL007C-E2 to create the plasmids pLD*spo0A* (table S1).

Retargeted pMTL007C-E2 plasmids were transformed into the *E. coli* conjugation donor strain CA434 and transferred into *C. difficile* strain 630Δ*erm* or R20291 by conjugation as previously described [29]. Transconjugants were selected for with thiamphenicol (15 µg/ml final concentration, Sigma). Screening for chromosomal insertion of the intron was performed with lincomycin (20 µg/ml, Sigma-Aldrich) to select for the restored *ermB* retrotransposition-activated marker (RAM) that signals integration into the genome. DNA was extracted from lincomycin resistant, thiamphenicol sensitive colonies (to indicate loss of the plasmid). A complement was constructed for R20291_*spo0A*::CT, labeled R20291_*spo0A*::CT::p*spo0A* [24] (table 1).

### Screening of the mutant by PCR and verification of mutants

Potential mutants were screened by PCR, sequencing and Southern blot analysis to confirm the chromosomal integration of the intron within the desired genes and loss of the plasmid pMTL007C-E2. Three PCRs were performed to screen putative mutants using the following oligonucleotides (table S1): i) RAM-F and RAM-R, to screen for loss of the group I intron, ii) a gene specific primer and the group II intron specific EBS universal primer, and iii) gene specific forward and reverse primers that flank the insertion site. The thermal cycling conditions were as follows: 95°C for 2 min×1; 95°C for 30 sec, 50°C for 30 sec, 68°C for 8 min×35 cycles; and 68°C for 10 min×1.

Sequencing was performed across the junction of the gene to intron using gene specific primers and the EBS universal primer to verify insertion site. Southern blot analyses were performed using AlkPhosDirect™ Labelling and detection kit (GE Healthcare) and detection reagents, in accordance with the manufacturer's guidelines and visualised using CDP star (GE Healthcare). Genomic DNA from wild type and potential mutants was digested with *BbsI* (Southern band size for the mutant 6314 bp). The probe was produced by PCR using RAM F and R primers (table S1), from within the group II intron sequence.

### Viability counts from the biofilms

The relative proportion of viable vegetative cells and spores was determined from three and six day old biofilms grown in static TC flasks (BHIS broth) in triplicate. The three or six day old biofilms were disrupted by vortexing and serial dilutions in 1× PBS were plated in triplicate onto BHIS plates supplemented with 0.1% taurocholate. In order to enumerate the number of spores present, 1 ml of disrupted biofilm was heated to 65°C for 25 minutes, killing the vegetative cells, and plated in triplicate onto BHIS agar plus 0.1% taurocholate. After 24 hour incubation, the CFU/ml was determined. The disruption of the biofilm, the serial dilutions and plating was performed under anaerobic conditions. The number of spores was subtracted from the total cell counts to give the vegetative cell numbers. All experiments were performed in quadruplicate. These were plotted graphically in Prism 4.0. A Chi-squared (χ^2^) interaction test was used to determine if any differences observed between the stains were dependent on the age of the biofilm (three days and six days). A regression analysis was then performed to determine a) were there significant differences in the number of i) vegetative cell or ii) spores between strains, in three day old biofilms? b) were there specific differences in the number of i) vegetative cells or ii) spores between strains in six day old biofilms? c) were there significant difference in the number of viable cells present in six day old biofilms compared to three day old biofilms? (table 2). For all the analyses *p*<0.05 indicates a significant difference and the coefficient (C) determines whether this difference is higher (positive number) or lower (negative number) than the reference (indicated in table 2) (C = 0).

**Table 2:** The relative survival of vegetative cells and spores in three day old and six day old biofilms.

			Regression analysis	
Category	Cell type	Strains	coefficient (C)	*p-*value
Three day old biofilm	Vegetative cells	R20291	0	reference
		630Δerm	**−0.412**	**0.044**
		spo0A mutant	0.326	0.078
		spo0A complement	**0.377**	**0.049**
	Spores	R20291	0	reference
		630Δerm	**−1.636**	**0.000**
		spo0A mutant	**−11.486**	**0.000**
		spo0A complement	**−3.231**	**0.000**
Six day old biofilm	Vegetative cells	R20291	0	reference
		630Δerm	−0.477	0.099
		spo0A mutant	0.319	0.247
		spo0A complement	**0.594**	**0.024**
	Spores	R20291	0	reference
		630Δerm	**−2.829**	**0.000**
		spo0A mutant	**−12.715**	**0.000**
		spo0A complement	**−3.397**	**0.000**
Category	Cell type	Biofilm	coefficient (C)	*p-*value
Comparison	Vegetative cells	Three day	0	reference (R20291_day 3)
		Six day	**−3.461**	**0.000**
	Spores	Three day	0	reference (R20291_day 3)
		Six day	**0.675**	**0.000**

A regression analysis was performed to determine whether there were any strain specific differences in the levels of vegetative cells and spores at three days compared to the R20291 reference. This was repeated for the six day old biofilms. *p*<0.05 indicates a significant difference from the reference R20291. The COV indicates whether the difference in spore and vegetative cell production is higher (positive number) or lower (negative number) compared to the R20291 reference. A regression analysis was also used to determine whether there were significant changes in the cell type from three days to six days, *p*<0.05.

### Oxygen stress assay

Biofilms were produced in BHIS media in TC flasks as outlined above. At three days post inoculation duplicate cultures were subjected to oxygen stress for 24 hours. Control biofilms were incubated for an additional 24 hours under anaerobic conditions. CFU assays were then performed under anaerobic conditions as outlined above and the vegetative cell and spore survival determined. All assays were performed in triplicate. The data was analysed in excel and graphs were plotted in Prism 4.0. Regression analysis was performed to answer the following questions a) were there significant differences between the strains in i) untreated biofilms, ii) intact stressed biofilms and iii) disrupted stressed biofilms, b) were there significant differences in the number of i) vegetative cells or ii) spores in the intact stressed biofilms compared to the untreated controls, c) were there significant differences in the number of i) vegetative cells or ii) spores in the disrupted stressed biofilms compared to the untreated controls and d) were there significant differences in the number of i) vegetative cells or ii) spores in the disrupted stressed biofilms compared to the intact stressed biofilms. *p*<0.05 indicates a significant difference between strains and the coefficient (C) determines whether this difference is higher (positive number) or lower (negative number) than the reference (R20291) (C = 0).

## Results

### Biofilm formation on abiotic surfaces

**Figure 1:**
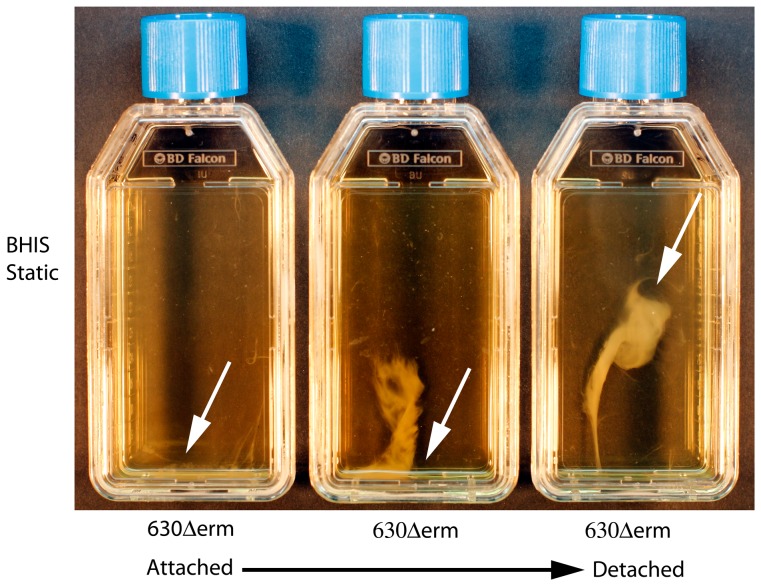
Formation and visualisation of 3 day old biofilms attached to an abiotic surface. The attachment of *C. difficile* strain 630Δerm is first visible at 16–24 hours, after which it matures, and can be visualised at the bottom of a static tissue culture flask (day 3). This attachment to the flask can be disrupted by gentle agitation, upon which the biofilm detaches from the plastic, but remains intact in the liquid media. The white arrows indicate the biofilm, and the black arrow indicates the transition from attached to detachment of the biofilm.

**Figure 2:**
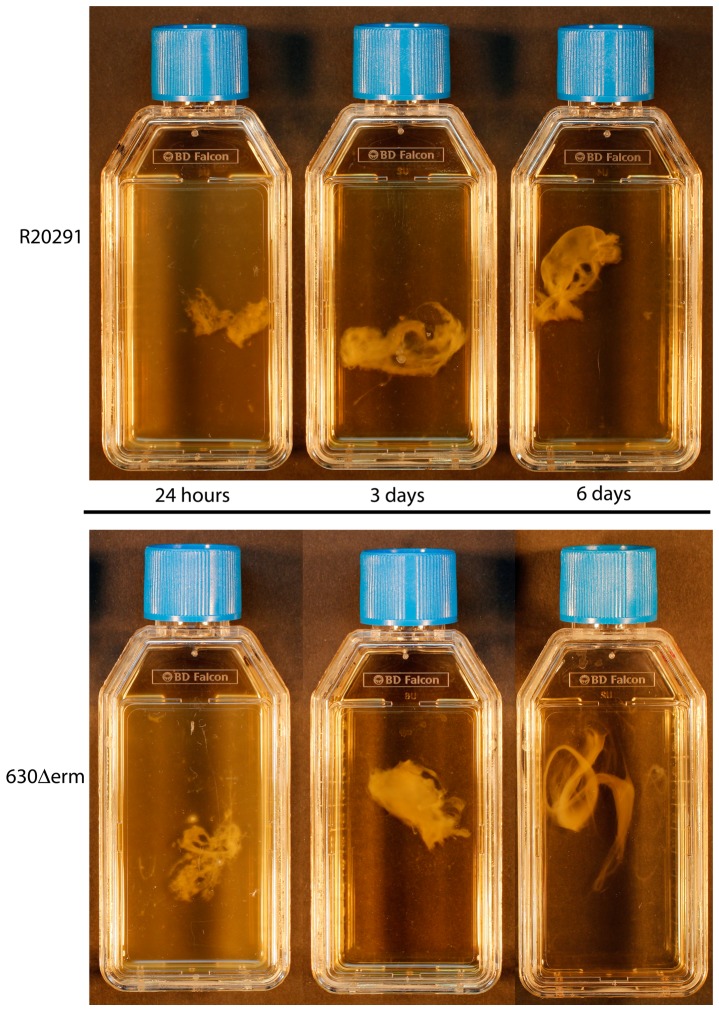
Maturation of the *C. difficile* biofilm from 24 hours to six days. The biofilms observed in the tissue culture flasks were produced after incubation for either; 24 hours, three days or six days, before they were mechanically detached from the plastic by agitation as outlined in figure 1. The top panel corresponds to the biofilm mass observed in strain R20291 and the bottom corresponds to 630Δerm.

The aggregation of *C. difficile* strains 630Δ*erm* (derived from the PCR ribotype 012 sequenced strain 630) and R20291 (PCR ribotype 027) was observed over a six day period. Sedimentation began at the bottom of a statically incubated TC flask from 16 hours post inoculation, these microcolonies matured, and biofilms were visualized at three days attached to the bottom of the TC flask (figure 1). The attached biofilm could be detached gradually from the plastic by agitation as a single mass for visualization (figure 1). The formation and maturation of the biofilms was gradual, as the static cultures entered stationary phase (12 hours), the media was turbid, however from 16–24 hours the turbidity of the media decreased and the size of the aggregate attached to the plastic TC flask visually increased (figure 2). The size of the biofilm mass appears to increase visually from 24 hours to six days for both *C. difficile* strains 630Δ*erm* and R20291 (figure 2). This was observed by detaching the biofilm as a single mass from the bottom of the plastic TC flask, by agitation (figure 2).

**Figure 3:**
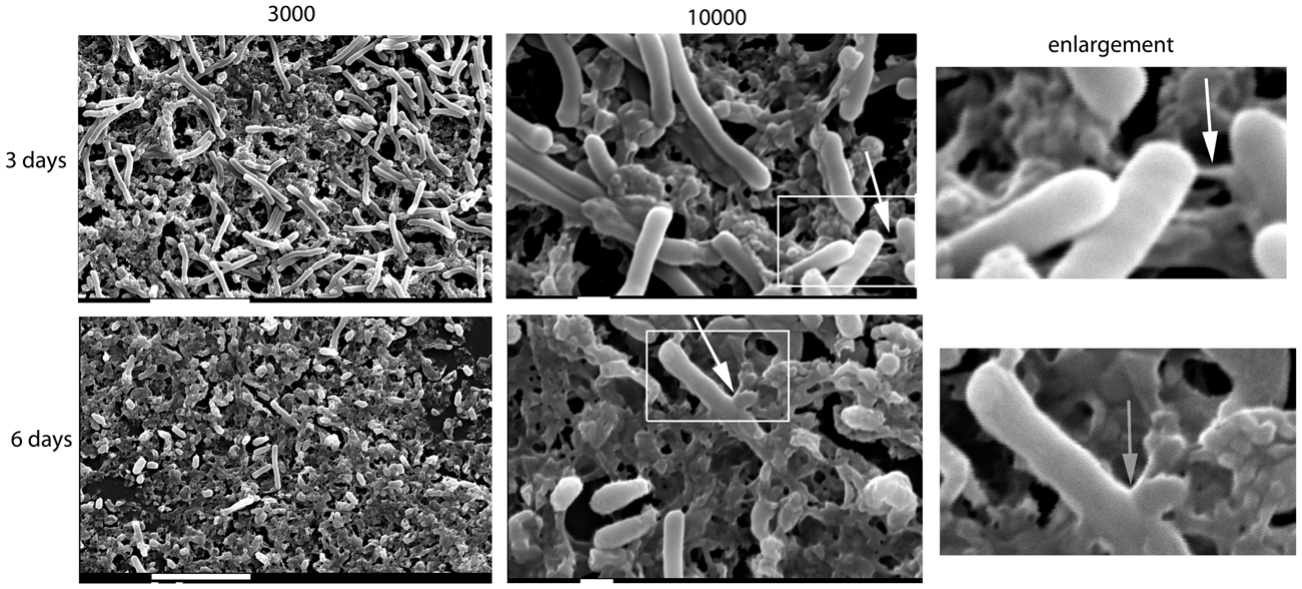
SEM visualisation of biofilm. The bacteria and the EPS matrix were visualised by SEM at ×3000 and ×10000 magnifications. The top panel corresponds to three day old biofilms and the bottom panel to six day old biofilms. The white boxes indicate the enlarged sections, and the white arrow indicates the connections between bacteria in three day old biofilms and in the six day old biofilms the white arrow shows the thicker matrix attached to the bacteria. Scale bar = 10 µm for the ×3000 magnification and 1 µm for the ×10,000 magnification.

The biofilms produced by *C. difficile* strain R20291, attached to an abiotic surface (Thermanox coverslips) were also visualized at three and six days by SEM (figure 3). The ×3000 images at three and six days reveal a mat of biofilm across the coverslip. The bacteria (rods) and the EPS matrix were observed in three and six day old biofilms, however, there were more visible vegetative cells (rods) at three days compared to six days (figure 3). Connections between the bacteria and the matrix can be observed at ×10,000 magnification in the three day old biofilm (figure 3, top panel, white arrows) and at six days, however the bacteria appear to be more encapsulated within the matrix at six days (figure 3, bottom panel).

### Viability of the cells within a biofilm

**Figure 4:**
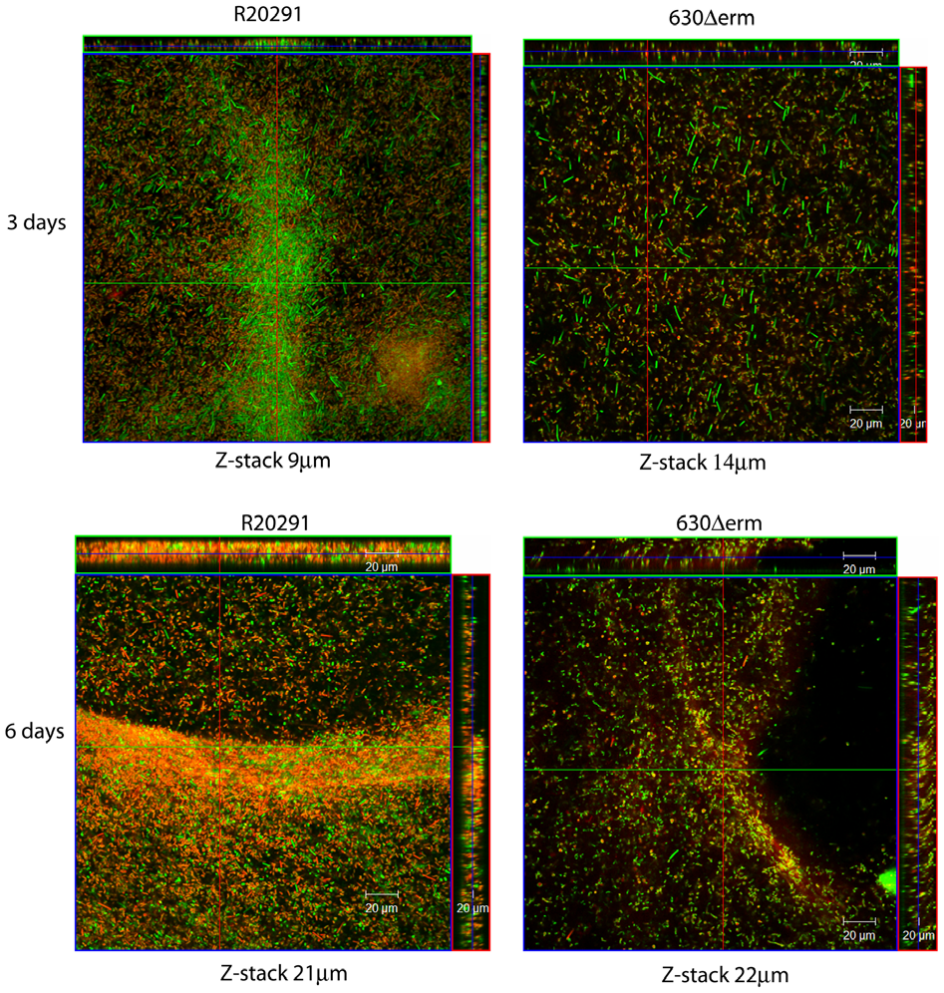
Confocal microscopy staining of three and six day old biofilms. Biofilms attached to plastic coverslips were stained using the FilmTracer™ LIVE/DEAD® biofilm viability stain. The images are Z-stack projections indicating the thickness of the biofilms for strain R20291 and 630Δerm for both three day and six day old biofilms. The depth of the Z-stack is indicated below the images in µm. These are representative Z-stacks from three independent replicates.

The viability of cells attached to an abiotic surface (coverslips) within the three-day old and six-day old biofilms, were assessed using FilmTracer™ LIVE/DEAD® biofilm viability staining and visualized by confocal microscopy. More live cells were observed in the three-day biofilms compared to the six-day biofilms in both strains (figure 4). Z-stack projections indicate that the biofilms were generally thicker at six days post seeding for both strains, 21 µm (R20291) and 22 µm (630Δ*erm*) compared to 9 µm (R20291) and 14 µm (630Δ*erm*) at day 3 (figure 4). However, the depth of the biofilms varied across the coverslip.

### Visualisation and integrity of biofilm formation in Spo0A mutant

**Figure 5:**
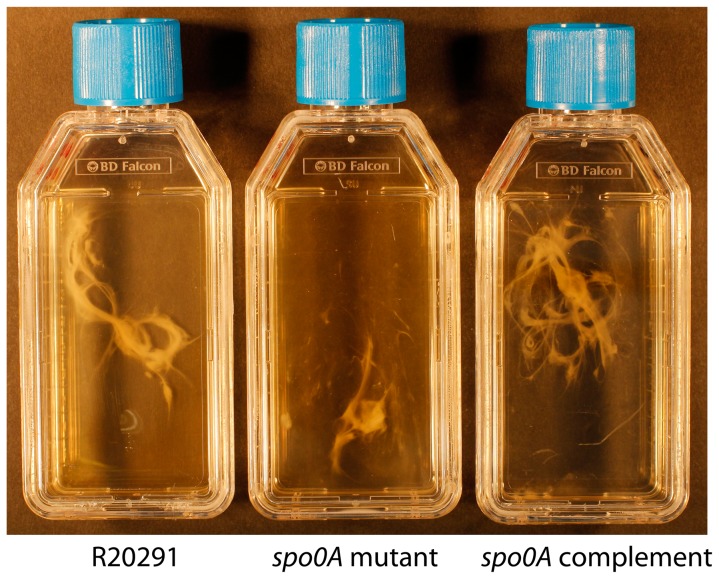
The formation and visualisation of three day old biofilms in the *spo0A* mutant. The biofilms produced attached to a TC flask were compared for R20291, R20291_*spo0A*::CT and R20291_*spo0A*::CT::*pspo0A*. These attached biofilms were matured over a three day period, before they were detached from the plastic by agitation for visualisation and comparison.

To determine if Spo0A is also involved in *C. difficile* biofilm formation a *spo0A* knock-out mutant (R20291_*spo0A*::CT) was generated by ClosTron mutagenesis and complemented [30], [31] (table 1). The aggregation of R20291, *spo0A* mutant (R20291_*spo0A*::CT) and complement (R20291_*spo0A*::CT::p*spo0A*) and the formation of biofilms was observed over a six-day period in TC flasks. Sedimentation of the bacteria to the bottom of the TC flask had begun at sixteen hours post seeding, similar to the other strains analysed (figure 1 and 2). Over the six-day period, the turbidity of the media decreased and the size of the aggregate visually increased. However, upon gentle agitation, the *spo0A* mutant strain detached and disbursed more readily from the plastic, than the wild-type and complement (R20291_*spo0A*::CT::p*spo0A*). The biofilm was a smaller, more disbursed mass in the liquid media compared to R20291 and the *spo0A* complement (figure 5), with some turbidity in the media.

**Figure 6:**
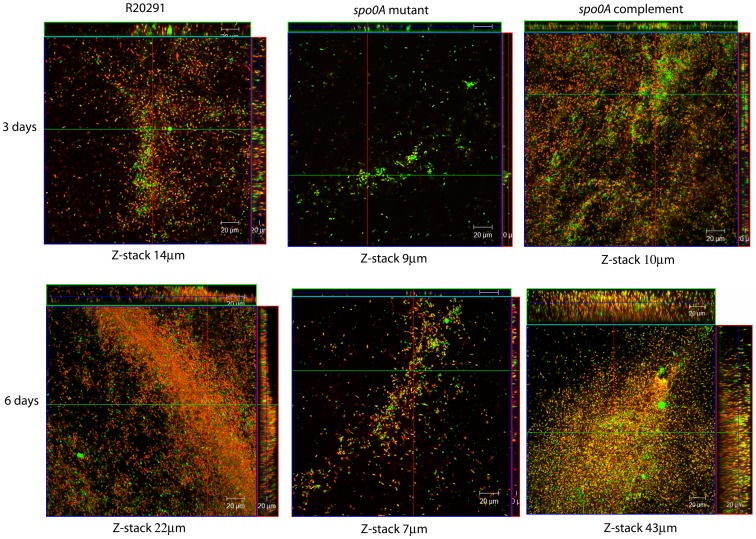
Confocal microscopy of biofilms attached to an abiotic surface. Biofilms were matured over a three day and six day period attached to Thermanox coverslips in BHIS media. These were then stained with the FilmTracer™ LIVE/DEAD® biofilm viability stain to assess the viability of the cells within the biofilm. Z-stack projections are representatives from three independent replicates which indicate the depth of the biofilms for each strain, R20291, R20291_*spo0A*::CT and R20291_*spo0A*::CT::*pspo0A* at both three and six days post seeding.

The composition of the biofilms attached to an abiotic surface (Thermanox coverslip) was assessed using the FilmTracer™ LIVE/DEAD® biofilm viability Kit (Life Technologies) (figure 6). The biofilm produced by the *spo0A* mutant is considerably smaller than the wild type and complement strain when visualized by confocal microscopy (figure 6). The Z-stack projections show a reduction in the depth and breadth of the biofilms produced in the *spo0A* mutant (figure 6). This visual difference is consistent at three days and six days, by both confocal microscopy (figure 6) and in TC flasks (figure 5 and data not shown). The Z-stack projections indicate that the general depth of the biofilm increases in all the strains from three to six days with the exception of the *spo0A* mutant. The depth of the R20291 biofilm increased from 14 µm to 22 µm. The *spo0A* complement increased from 10 µm to 43 µm, whereas the spo0A mutant decreased slightly from 9 µm to 7 µm. It is clear that the R20291_*spo0A*::CT::p*spo0A* strain is complementing the reduced biofilm phenotype, however there are subtle differences in the FilmTracer™ LIVE/DEAD® biofilm viability Kit straining between the R20291 parent strain and R20291_*spo0A*::CT::p*spo0A*. There appear to be more live/living cells in the R20291_*spo0A*::CT::p*spo0A*, and more dead/dying cells in the wild-type R20291, particularly at day six (figure 6). One explanation is that the spores appear to stain predominantly red (figure 6, R20291), suggesting that the complementation of Spo0A is restoring the biofilm phenotype (as seen by the Z-stack projection), but only partially restoring the sporulation phenotype (as seen by the proportion of live/dead staining).

### Quantification of biofilm formation

**Figure 7:**
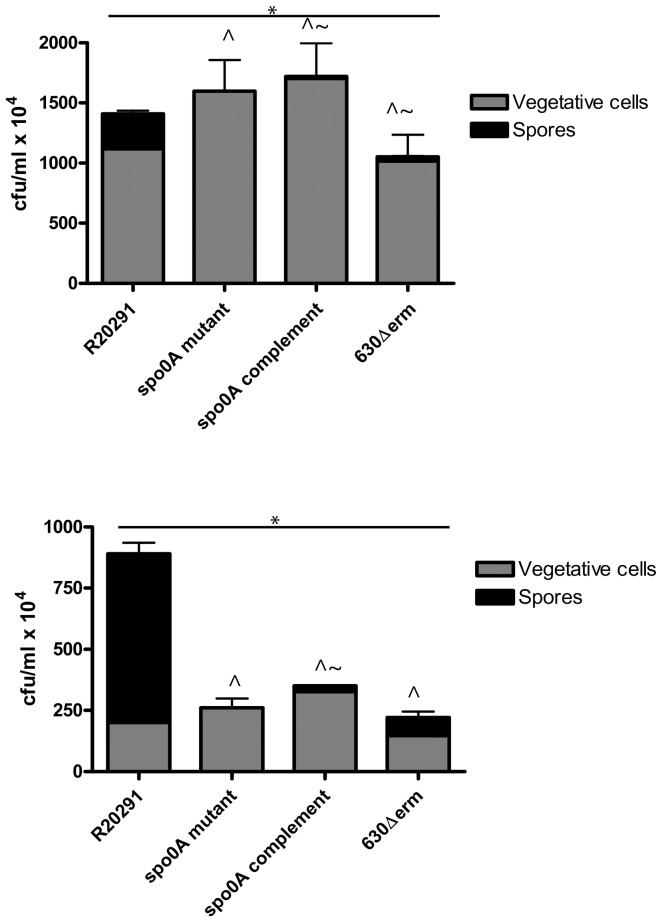
Enumeration of vegetative cells and spores encased within a biofilm. Biofilms were matured for three and six days post seeding in TC flasks for strains R20291, R20291_*spo0A*::CT, R20291_*spo0A*::CT::p*spo0A* and 630Δerm. The biofilms were detached from the plastic and dispursed by vortexing. These suspensions were used to determine the proportion of vegetative cells and spores per ml of biofilm (10 ml total volume). A partial F-test revealed strain specific differences at three days and six days, indicated with * (*p*<0.001). Regression analysis was used to determine whether there were significant differences in the levels of spores and vegetative cells at each time point, compared to the R20291 reference, as indicated by ∧ for spores (*p*<0.05) and ∼ for vegetative cells (*p*<0.05). Differences between the cell types in six day old biofilms compared to three day old biofilms are listed in table 2.

The proportion of vegetative cells and spores within the biofilm were quantified in triplicate by CFU counts, for three and six day old biofilms. The biofilms contain a large number of vegetative cells and spores, for example R20291 contains an average of 1.6×10^7^ vegetative cells and 2.3×10^6^ spores for R20291 per three day old biofilm (CFU/ml), and 1.7×10^6^ vegetative cells and 7.0×10^6^ spores per six day old biofilm (CFU/ml) (figure 7). The number of spores present in the cultures increased over time, in the primary 16 hour old cultures used to seed the biofilms there were an average of 1.8×10^3^ spores (CFU/ml) for R20291 and for 630Δerm they were below the limit of detection of the assay, therefore <1×10^2^ spores (CFU/ml). Whereas, in 24 hour old secondary cultures, there were 5.7×10^4^ (CFU/ml) for strain 630 and 4.6×10^4^ (CFU/ml) for strain R20291 [33]. Statistical analysis was performed to determine the strain specific differences in cell type observed in the three day old and six day old biofilms. A partial F-test revealed that there are strain specific differences in the levels of spores and vegetative cells (*p*<0.05) in three day old biofilms. A regression analysis revealed that strains R20291_*spo0A*::CT, 630Δerm and R20291_*spo0A*::CT::p*spo0A* have significantly reduced numbers of spores compared to R20291 (*p*<0.05) in both 3 day old and 6 day old biofilms (table 2). Strain 630Δerm contains less vegetative cells compared to R20291 (*p*<0.05) (consistently in both 3 day old and 6 day old biofilms), whereas *spo0A* complement contains more vegetative cells than R20291 (*p*<0.05) (consistently in both 3 day old and 6 day old biofilms. This is consistent with the FilmTracer™ LIVE/DEAD® biofilm viability staining, where it appears that the complement contains more live/living cells than R20291. Interestingly, this indicates that despite the decreased biofilm formation in the *spo0A* mutant the overall number of viable cells in the culture is not reduced, however these viable cells are not encased within a matrix. A partial F-test revealed that there are strain specific differences in the levels of spores and vegetative cells (*p* = 0.0013) in six day old biofilms. A comparison (regression analysis) was performed between the three day and six day biofilms to determine whether there were strain specific differences in the numbers of viable cells; a) taking strain into consideration, there were significantly less vegetative cells at day three compared to day six *p*<0.01, and C = −3.461 (table 2) b) taking strain into consideration, there were significantly more spores at six days compared to three days *p*<0.01, C = 0.675 (table 2) (with the exception of the *spo0A* mutant, which does not sporulate). The most visible difference in biofilm composition was observed with R20291, in which levels of spores increased by 67% from three days to six days. The CFU data confirms our hypothesis from the FilmTracer™ LIVE/DEAD® biofilm viability staining that although the biofilm phenotype is restored, the sporulation phenotype is only partially complemented by *spo0A* carried on a plasmid in strain R20291_*spo0A*::CT::p*spo0A*.

**Figure 8:**
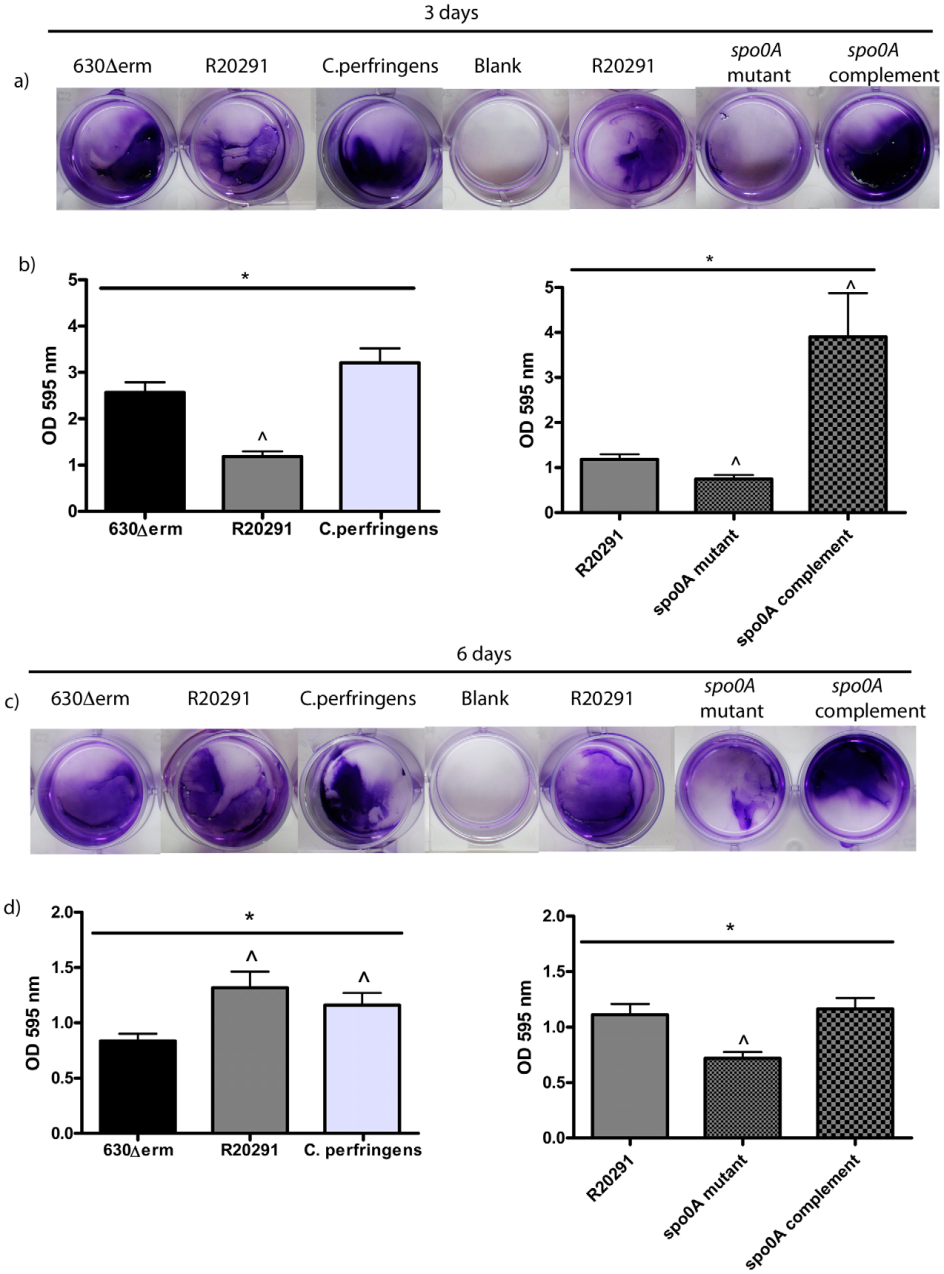
Crystal violet quantification of biofilm formation. a) Visualisation of three day old biofilms attached to the bottom of the individual wells in a 24 well plate, compared to a media blank control. b) Graphical representation of the optical density readings OD595 nm derived from methanol elution of the crystal violet stain. A partial F-test was performed to determine if there were strain specific differences in the level of biofilm formation, indicated with * (*p*<0.05). A regression analysis was performed to determine whether there were any specific strain differences compared to the reference indicated with ∧ (*p*<0.05). The reference was 630Δerm for the first graph, and R20291 for the *spo0A* mutant and wild-type comparison. c) Visualisation of six day old biofilms attached to the bottom of a 24 well plate as outlined in (a). d) Graphical representation of the optical density readings OD595 nm derived from methanol elution of the crystal violet stain, as outlined in (b). Statistical analysis was performed as outlined above.

In addition to determining vegetative cell and spores counts, crystal violet assays were performed to quantify the level of biofilm formation between strains. A comparison was performed between the crystal violet OD readings for three and six day old biofilms (figure 8). Three day old biofilms attached to the bottom of the well in a 24-well plate, were visualised by staining with crystal violet (figure 8a) and quantified (OD595 nm) by methanol extraction (figure 8b). The bottom panel shows the equivalent at six days (figure 8c and d). The crystal violet stain reveals visual differences in the size of the biofilm between strains (figure 8a and c). The level of biofilm formation is consistently reduced in the *spo0A* mutant strain, at both three days and six days. Statistical analysis was performed on the crystal violet assays to determine if there are strain specific differences in biofilm formation at three and six days. A Chi-squared interaction test (χ^2^) was performed to determine whether incubation period (days) and biofilm formation (OD) were mutually exclusive. There is strong evidence (*p*<0.01) that both incubation period (day) and strain effect the biofilm formation (OD) (χ^2^ *p* = 0.000). A partial F-test revealed strong evidence (*p* = 0.000) that there were differences in biofilm formation between strains. To pinpoint the strain specific differences in biofilm formation, regression analysis was performed on the strains separated into two groups, a) three day biofilms, b) six day biofilms. a) At three days, 630Δ*erm* (*p* = 0.002, C = 0.762), *C. perfringens* (*p* = 0.000, C = 1.023) and the *spo0A* complement (*p* = 0.008, C = 0.630) produced significantly more biofilm than R20291 (table 3), whereas the *spo0A* mutant produced significantly reduced (*p*<0.05) biofilm (*p* = 0.020, C = −0.523) (table 3, figure 8b). b) At six days, regression analysis strongly indicates that 630Δ*erm* (*p* = 0.008, C = −0.339) and R20291_*spo0A*::CT (*p* = 0.000, C = −0.468) form less biofilm than R20291 (table 3 and figure 8d). The *spo0A* mutant produces lower levels of biofilm than R20291 at both three days and six days. (table 3 and figure 8b and d).

**Table 3:** The quantitation of biofilm formation by crystal violet in three day old and six day old biofilms.

		Regression analysis	
Category	Strains	coefficient (C)	*p-*value
Three day old biofilm	R20291	0	reference
	630Δerm	**0.762**	**0.002**
	*C. perfringens*	**1.023**	**0.000**
	spo0A mutant	**−0.523**	**0.020**
	spo0A complement	**0.629**	**0.008**
Six day old biofilm	R20291	0	reference
	630Δerm	**−0.339**	**0.008**
	*C. perfringens*	0.029	0.839
	spo0A mutant	**−0.468**	**0.000**
	spo0A complement	−0.007	0.954

A regression analysis was performed to determine whether there were any strain specific differences in the levels of biofilm formation at three days compared to the R20291reference. This was repeated for the six day old biofilms. *p*<0.05 indicates a significant difference from the reference R20291. The COV indicates whether the difference in biofilm formation is higher (positive number) or lower (negative number) compared to the R20291reference. A regression analysis was also used to determine whether there were significant changes in the biofilm formation from three days to six days, *p*<0.01.

### Resistance to oxygen stress

**Figure 9:**
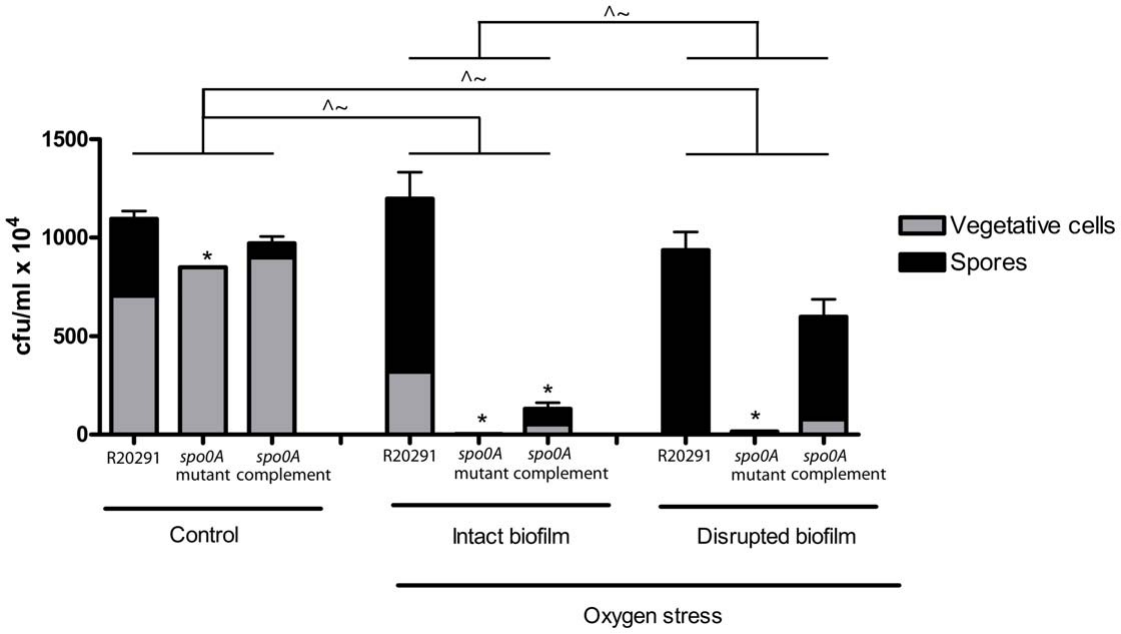
Relative survival of vegetative cells and spores within an intact and disrupted biofilm exposed to oxygen stress. The ability of a biofilm to protect against oxygen stress was assessed in three day old biofilms exposed to oxygen stress for 24 hours, alongside oxygen stress disrupted biofilms (vortexed). The survival is expressed as cfu/ml ×10^4^. Regression analysis was performed to determine if there were any significant differences in total cell counts between the wild-type, *spo0A* mutant and complement for the control group, intact biofilms and the disrupted biofilms (p<0.05 indicated with *). Additional regression analyses were performed to determine whether there were significant differences in the number of vegetative cells (∼) and spores (∧) in the intact and disrupted biofilms compared to the untreated controls (*p*<0.05). Finally analysis was performed to determine whether the numbers of vegetative cells (∼) and spores (∧) were significantly altered in the disrupted biofilm compared to the intact biofilm (*p*<0.05).

To determine whether the biofilm provides a protective environment and thus contributes to *C. difficile* survival, a stress assay was performed, looking at the response to oxygen stress. A regression analysis revealed that there is strong evidence the oxygen stress has a significant impact on the survival of the *spo0A* mutant (*p*<0.001), in intact and disrupted biofilms compared to the untreated R20291 (table 4, section a). The survival of the complement was not significantly different in disrupted biofilms compared to R20291, but the complementation was incomplete in the intact biofilms, resulting in a decrease of viable cells compared to R20291 (table 4, section a). Further analysis was performed to determine if there were differences in the cell types (spores/vegetative cells) present in the intact and disrupted biofilms. The survival assay indicated an increased number of spores present in intact biofilms in response to oxygen stress in all strains (p<0.05, C = 0.55) except the *spo0A* mutant, which does not produce spores. This increase in spore numbers is mirrored by a decrease in the number of vegetative cells across all the strains tested (*p*<0.001, C = −6.89) (table 4, section b) (figure 9). However, vegetative cell survival remained at 45% for R20291 and 630Δ*erm* for 66% (data not shown), respectively in intact biofilms stressed with oxygen. In contrast, when the vegetative cells are not protected by an extensive biofilm, as observed in the disrupted biofilms, the level of vegetative cells was below the limit of detection for R20291 (figure 9) (table 4, section c). There was an extremely low level of survival (<0.5%) of the vegetative cells, in the *spo0A* mutant in both the intact and disrupted biofilms (figure 9). This data indicates that intact biofilms have the potential to protect R20291 vegetative cells from external stress.

**Table 4:** The effect of oxygen stress on viability of cells within a biofilm.

Test Variable	Stress	Biofilm	Strain	Coefficient (C)	*p*-value
a) Strain	Untreated	Intact	R20291	0	reference
			spo0A mutant	**−5.72**	**0.000**
			spo0A complement	−0.75	0.446
	Oxygen stress	Intact	R20291	0	reference
			spo0A mutant	**−9.41**	**0.000**
			spo0A complement	**−3.86**	**0.001**
		Disrupted	R20291	0	reference
			spo0A mutant	**−4.07**	**0.000**
			spo0A complement	−0.04	0.964

Test Variable	Stress	Cells	Biofilm	Coefficient (C)	*p*-value
b) Cell type	Oxygen stress	Vegetative cells	R20291_untreated	0	reference
			Intact	**−6.89**	**0.000**
			disrupted	**−10.78**	**0.000**
		Spores	R20291_untreated	0	reference
			Intact	**0.55**	**0.013**
			disrupted	**1.19**	**0.000**

Test Variable	Stress	Cells	Biofilm	Coefficient (C)	*p*-value
c) Biofilm	Oxygen stress	Vegetative cells	Intact	0	reference
			Disrupted	**−3.96**	**0.002**
		Spores	Intact	0	reference
			Disrupted	**0.69**	**0.01**

Regression analyses were performed in three categories; a) were there strain dependent differences in survival in both the intact, disrupted and control biofilms. b) were there any significant differences in survival of vegetative cells or spores in intact and disrupted biofilms compared to the untreated control and c) were there any differences in vegetative cell or spore survival in intact biofilms compared to disrupted biofilms in response to oxygen stress. *p*<0.05 is significant (in bold).

## Discussion

A number of clinically and economically important bacteria possess the ability to form complex architectural communities comprising of cells held together with an EPS matrix scaffold. These structures, known as biofilms, enable clinically important pathogens such as *Staphylococcus aureus* and *Pseudomonas aeruginosa*, to colonise a particular niche, enhance bacterial survival and provide protection from environmental factors such as antimicrobials. The important nosocomial pathogen *Clostridium difficile* has been shown to form mats *in-vivo*, potential biofilms, during long-term colonisation in mice [34]. *C. difficile* has also been shown to form aggregates when the cell surface protein CwpV is constitutively expressed [35] and c-di-GMP, a known inducer of biofilm formation in *B. subtilis*, is present [36]. In this study, we demonstrate for the first time that *C. difficile* aggregates to form biofilms on abiotic surfaces. The *C. difficile* biofilm develops over time, after an initial sedimentation of bacteria at 16–24 hours. The composition and integrity of the biofilm matures over a six day period. Visualisation of the biofilm by SEM shows attachment of the biofilm to an abiotic surface, and elucidates the architecture of the bacterial communities encased within an EPS matrix. The confocal Z-stack projections give an indication of the depth of the biofilms, and the FilmTracer™ LIVE/DEAD® biofilm viability staining visually indicates the presence of both viable and dead/dying bacteria contained within an EPS matrix. The hypervirulent strain R20291, responsible for the outbreak in Stoke Mandeville in 2006, appears to produce a more viable biomass comprised of both vegetative cells and spores than the 630Δ*erm*. At six days, the total number of viable cells (spores capable of germination and vegetative cells) exceeded 8.6×10^6^/ml for R20291. One striking feature of the *C. difficile* biofilm is the ability to detach from the abiotic surface by agitation, to produce a single mass, or free floating aggregate, which may have ramifications in treatment of CDI.

SEM analysis of the *C. difficile* biofilms enabled visualisation of the bacteria encased within an EPS matrix and highlighted the filamentous connections within this biomass. Purcell and colleagues showed that elevated levels of c-di-GMP enhanced aggregation of *C. difficile* [36], which is analogous to the effects of c-di-GMP on *B. subtilis* biofilm formation. Their hypothesis was that c-di-GMP may upregulate expression of pili to enhance aggregation of the bacteria, given that pili have been shown to be involved in biofilm formation in other organisms. However, the connections between bacteria and the EPS matrix observed in this study are thicker and more defined than the ultra-fine structures observed in *C. difficile* aggregates by Purcell and colleagues [36]. In another study, Donelli and colleagues showed that *C. difficile* was capable of forming dual species biofilms, although in their study *C. difficile* did not form monospecies biofilms on an abiotic surface after 24 hours [37]. However, given the media, changing morphology and mass of the biofilms observed in this study over a six day time course, it is possible that media and incubation time were a factor in Donelli and colleagues being unable to detect a monospecies biofilm at 24 hours [37].

Regulation of biofilm formation and sporulation appear to be closely linked and tightly regulated in *B. subtilis* [17]–[20], by Spo0A. In *B. subtilis*, this master regulator can drive one of three pathways, sporulation, matrix production and cannibalism, all regulated by differential phosphorylation of Spo0A [38]. Low level phosphorylation of Spo0A indirectly induces expression of matrix inducing genes, such as the exopolysaccharide biosynthesis genes (*epsA-O*) [18] and the amyloid fibres produced by expression of *tasA* (*yqxM-sipW-tasA*) [39], [40], via the repressor and agonist SinR/SinI [20]; whereas, high level phosphorylation of Spo0A leads to the irreversible induction of the sporulation cascade, thus deciding the fate of a bacterium [18].

The role for Spo0A in biofilm formation in *C. difficile* was assessed in this work. The quantity of biofilm produced in the R20291_*spo0A*::CT strain is visually significantly reduced compared to the R20291 parent strain attached to TC flasks and plastic coverslips. The Z-stack measurements indicate approximately 36% less depth to the biofilm produced at three days and 69% less depth at six days, compared to the R20291 wild-type strain. These observations were upheld with the quantification by crystal violet staining and CFU assays. The CFU assays revealed there are strain specific differences in the number of viable cells (vegetative cells and spores) contained within a biofilm, and that the number of vegetative cells decreases from three to six days as the level of spores increase, across all the strains tested (except R20291_*spo0A*::CT which does not sporulate). The biofilms formed with the hypervirulent strain R20291, were consistently larger and contained more viable cells that the other strains tested. There was a shift in the cell type from three days to six days, there is a 3 fold increase in the number of spores, and a 10 fold decrease in the number of vegetative cells at six days compared to 3 days for R20291. Potentially there may be a yet unidentified signal to convert from the biofilm lifestyle to sporulation pathway, which may be involved in virulence and transmission of *C. difficile*. Complementation by a plasmid encoding a copy of *spo0A* reinstates the biofilm phenotype, as determined visually in the TC flasks and quantitatively by crystal violet, fluorescence microscopy and CFU; however, the sporulation phenotype is only partially restored. The CFU assays revealed low levels of sporulation compared to R20291 wild-type. The complementation of the biofilm phenotype indicates that the level of spores is not essential for formation of biofilms, yet it indicates that the Spo0A regulator is important in the formation of biofilms. The partial complementation could be due to the intrinsic difficulties in attaining wild-type levels of expression of plasmid maintained genes. The decreased biofilm formation in *spo0A* mutant, suggests that like the gram positive bacterium *B. subtilis*, biofilm formation in *C. difficile* could be linked to sporulation, and regulated by Spo0A. *C. difficile* may have alternate pathways to sporulation, similar to the matrix production and cannibalism pathways in *B. subtilis*, which are involved in biofilm formation in *B. subtilis* and are all differentially regulated by phosphorylation levels of Spo0A.

This is the first evidence that the clinically important pathogen *C. difficile* forms biofilms on an abiotic surface. The visualisation of the biofilms by SEM gives an overview of the biofilm architecture, and the Z-stack projections provide evidence of the depth of these structures. The reduction of biofilm formation in the *spo0A* mutant and the increased sensitivity of this mutant to oxygen stress indicate that Spo0A is involved in production or maintenance of these biofilms. The biofilms are comprised of both spores and vegetative cells encased within an EPS matrix, which forms the scaffold structure and protective environment for enhanced survival to external stresses such as oxygen. In the absence of this protective biofilm, as seen in the disrupted R20291 biofilm and the *spo0A* mutant strain, the vegetative cells are extremely susceptible to oxygen stress. It is clear that without the protection of the biofilm, the vegetative cells from R20291 are highly susceptible to oxygen, however, deletion of the master regulator spo0A may have an indirect effect on tolerance to oxygen stress. The mechanism by which *C. difficile* colonises and progresses through the gut is unclear. Biofilm formation could be an important factor in the infection process and disease outcome as biofilms may provide a potential reservoir of bacteria (vegetative cells and spores) to re-colonise an individual after treatment and contribute to the high incidence of clinical relapse.

## Supporting Information

Table S1 Primers used in the study.
(DOCX)
